# Subjetividades promovidas por las políticas de alimentación y nutrición: el caso de la Comuna 1 Popular de Medellín, Colombia

**DOI:** 10.18294/sc.2025.5544

**Published:** 2025-04-25

**Authors:** Paula Andrea Giraldo-López, Lorena Mancilla-López, Luz Adriana Muñoz-Duque

**Affiliations:** 1 Magíster en Políticas Públicas Alimentarias y Nutricionales. Docente, Escuela de Nutrición y Dietética, Universidad de Antioquia, Medellín, Colombia. paula.giraldol@udea.edu.co Universidad de Antioquia Escuela de Nutrición y Dietética Universidad de Antioquia Medellín Colombia paula.giraldol@udea.edu.co; 2 Doctora en Salud Pública. Profesora, Escuela de Nutrición y Dietética, Universidad de Antioquia, Medellín, Colombia. lorena.mancilla@udea.edu.co Universidad de Antioquia Escuela de Nutrición y Dietética Universidad de Antioquia Medellín Colombia lorena.mancilla@udea.edu.co; 3 Doctora en Salud Pública. Docente, Facultad de Ciencias Sociales y Humanas, Facultad Nacional de Salud Pública, Universidad de Antioquia, Medellín, Colombia. luza.munoz@udea.edu.co Universidad de Antioquia , Facultad de Ciencias Sociales y Humanas, Facultad Nacional de Salud Pública Universidad de Antioquia Medellín Colombia luza.munoz@udea.edu.co

**Keywords:** Protección Social en Salud, Programas y Políticas de Nutrición y Alimentación, Colombia, Social Protection in Health, Nutrition Programs and Policies, Colombia

## Abstract

El objetivo del estudio fue comprender las subjetividades promovidas por las políticas de alimentación y nutrición en la Comuna 1 Popular de Medellín. Desde un diseño cualitativo basado en la teoría fundamentada con enfoque constructivista, entre 2022 y 2023 se realizaron 20 entrevistas semiestructuradas a personas usuarias, lideresas sociales y a quienes implementan los programas alimentarios y nutricionales en la comuna. Entre los principales hallazgos se identificó que las políticas públicas alimentarias y nutricionales configuran subjetividades precarizadas, a través de mecanismos como la homogenización en la atención alimentaria y nutricional de las personas usuarias. Los aspectos administrativos de los programas limitan el desarrollo de acciones eficaces y la participación tiene baja incidencia política. Se concluye que la visión y alienación de sujetos individualizados limita las posibilidades de acción colectiva; sin embargo, las lideresas sociales promueven acciones para movilizar ajustes en el funcionamiento de estas políticas y programas.

## Abstract (en)

The aim of this study was to understand the subjectivities promoted by food and nutrition policies in Comuna 1 Popular of Medellín. Based on a qualitative design grounded in constructivist grounded theory, 20 semi-structured interviews were conducted between 2022 and 2023 with program users, social leaders, and those who implement food and nutrition programs in the comuna. Among the main findings, it was identified that public food and nutrition policies shape precarious subjectivities through mechanisms such as the homogenization of food and nutrition care for users. The administrative aspects of the programs limit the development of effective actions, and participation has low political impact. It is concluded that the vision and alienation of individualized subjects restrict the possibilities for collective action; however, social leaders promote actions aimed at mobilizing adjustments in the functioning of these policies and programs.

## INTRODUCCIÓN

El tipo de sujetos que configuran las políticas públicas alimentarias existentes no constituye un tema de especial interés en dicho campo, tampoco se incluye este aspecto en la evaluación de estos instrumentos de planificación. Asimismo, la información disponible sobre los efectos de las políticas alimentarias se centra en parámetros biológicos y de cumplimiento de metas, por tanto, se expresa en un lenguaje preeminentemente cuantitativo, el cual es limitado para visibilizar la voz de las personas y su capacidad de agencia cuando interactúan con las políticas y programas alimentarios. Durante mucho tiempo, y en especial en las ciencias de la nutrición, las subjetividades han sido poco exploradas, lo que ha llevado a ignorar sus diferentes expresiones como fuentes para la construcción de acontecimientos sociales^1^.

En el caso de América Latina es preciso señalar que la implantación de la corriente neoliberal a partir de la década de 1990 produjo la transformación de los Estados de esta región y esta ideología permeó todos los sectores del desarrollo social, produciendo el desmantelamiento sistemático del exiguo Estado de bienestar^2^. Este viraje neoliberal, como lo denomina Muller, ocasionó que la globalización fuera el referencial, es decir, la nueva norma para la configuración de las políticas públicas, con una importancia creciente del “mercado”^3^.

En la era neoliberal la lucha del Estado contra la pobreza se circunscribe a la provisión de servicios mínimos, dirigidos a los segmentos de la población que no pueden obtenerlos mediante el mercado, o como lo describe Castel a los “individuos por defecto”, es decir, aquellos que carecen de los mecanismos o soportes para demostrar que tienen un mínimo de independencia social. Estos individuos reciben prestaciones inferiores a las que reciben aquellos incluidos en el mercado del trabajo y, según este autor, estas prestaciones tienen dos características: son condicionales, es decir, se otorgan a públicos que demuestran que están en déficit respecto al régimen común; y tienen una lógica de la contraprestación, porque “los beneficiarios deben colaborar con los dispositivos que les son propuestos para ayudarlos”^4^. El autor añade que estas nociones conducen a la progresión de una lógica mercantil en el campo social en la que nada se otorga si no hay contraprestación por parte del individuo, “nada de ayuda a quien no trate de ayudarse a sí mismo”. Es por ello que esto marca el triunfo del principio de la individualización y, por tanto, estamos presenciando una “sociedad de los individuos”^4^.

Por consiguiente, la función de los Estados modernos se centra en la configuración de sujetos que contribuyan al fortalecimiento del capitalismo. Entre los dispositivos para el control de estos sujetos, el Estado acude a las políticas y programas de salud pública, en especial, las alimentarias y nutricionales, mediante las cuales introduce unas prácticas moralizadoras y disciplinarias como lo plantea Foucault^5^, es decir, discursos, modos y prácticas que se instauran mediante la implementación de la política, con el propósito de moldear un tipo de sujeto, el cual asuma comportamientos que encajen en la visión de lo que se espera de su rol como usuario. En este orden de ideas, en el marco de un Estado que tiene una concepción de lo social limitada a la mitigación de la pobreza, la política social se configura en una asistencia social condicionada a la demostración de necesidad^6^. 

Uno de los instrumentos más reconocidos en este papel del Estado es la focalización de las acciones, por tanto, en este contexto emergen conceptos como las “focopolíticas”, que aluden a “las formas de controlar y regular la vida o los umbrales de ella, de un creciente grupo de poblaciones pobres”, influir sobre ellas y gobernarlas, las cuales se describen también como una de las nuevas formas de gubernamentabilidad neoliberal^7^. Algunas condiciones del curso de vida, como la gestación o algunas de sus etapas, son usadas para transmitir un mensaje a favor de la atención de solo unas porciones de la población (focalización), a partir de la imagen de personas que son altamente valoradas y, a su vez, las más necesitadas^8^. 

Para el caso del Estado colombiano, el modelo neoliberal se evidenció en un desmesurado favorecimiento al sector privado y a las importaciones, incluidas las de alimentos, que presentaron un importante aumento desde 1990 a partir de los tratados de libre comercio^9^. En los países de bajos ingresos o del sur global, esta lógica de libre comercio socava la economía local de subsistencia, tornándolos vulnerables a los cambios tecnológicos y de precios, y haciéndolos dependientes de productos vendidos en el mercado mundial^10^. Las implicaciones de estas dinámicas sobre la agricultura familiar y comunitaria podrían resumirse en un mayor e histórico rezago socioeconómico de los pequeños productores, lo cual desincentiva la producción de alimentos autóctonos y, consecuentemente, afecta la cultura alimentaria y favorece la implantación de un sistema alimentario con alta producción, distribución y consumo de productos ultraprocesados, que están estrechamente relacionados con el aumento de las enfermedades no transmisibles (ENT), y con la doble carga de la malnutrición^11^. 

Si bien los grupos con mayor poder adquisitivo podrían acceder a una dieta más variada o demandar productos orgánicos, gran parte de la población queda atrapada en lo que Otero y Pechlaner denominan una “dieta neoliberal”^11^. Por consiguiente, como lo describe Ioris, “debido a la acumulación de fallas estatales y de mercado, el hambre y la desnutrición no se distribuyen al azar, sino que se concentran en ciertas áreas y entre ciertos grupos”^12^. 

Enmarcadas en estas formas de distribución de bienes y servicios y cargas sociales, emergen formas de ser y de experimentar las realidades en la vida cotidiana, toda vez que la característica propia del relacionamiento humano implica la producción de subjetividades en los diferentes espacios sociales e históricos en los que convergen. Las subjetividades constituyen las formas de explicitar un sistema complejo que permite trascender la fragmentación de comportamientos concretos en diferentes esferas de la vida de los sujetos, que se desarrollan dentro de un universo de realidades^1^, Bourdieu^13^ reafirma la perspectiva histórica del sujeto al indicar su idea de *habitus* como capacidad de generar, con una libertad controlada, pensamientos, percepciones, expresiones y acciones, que siempre tienen como límite las condiciones históricas y sociales en las que se sitúa su producción.

En el caso concreto de la Comuna 1 Popular, que se encuentra ubicada en la zona nororiental de la ciudad de Medellín en Colombia, es importante reconocer que el proceso de urbanización lo iniciaron, en la década de 1960, pobladores víctimas de la violencia, y durante la década de 1980 se consolidó como un sector receptor de población de bajos recursos económicos, situación que no ha cambiado en la actualidad^14^. Para 2020, en la Comuna 1 Popular, el 74% de los hogares se encontraban en inseguridad alimentaria, lo que la ubicaba como la comuna con mayor inseguridad alimentaria en la ciudad. Sus habitantes sobrevivían sobre la base de una economía principalmente informal y un gran número de mujeres como cabeza de familia^14^. En este contexto se implementaron políticas, programas y proyectos alimentarios y nutricionales del orden nacional y local. Por lo anterior, el objetivo del presente estudio fue comprender las subjetividades promovidas por las políticas de alimentación y nutrición en las personas usuarias, lideresas sociales e implementadoras de programas y proyectos alimentarios y nutricionales de la Comuna 1 Popular de Medellín.

## MÉTODOS

Este trabajo se inscribe en el enfoque interpretativista, corriente que hace parte del campo de análisis de las políticas públicas. Desde esta vertiente se opta por la perspectiva epistemológica histórico-hermenéutica, reconociendo la diversidad y la importancia de la participación para acercarse a la compresión de motivaciones, significados, cosmovisiones e intenciones, en el contexto de los enfoques usados para los programas en alimentación y nutrición, sin perder una postura crítica^15^. 

La selección de los participantes del estudio se realizó a través de la técnica de bola de nieve^16^. A partir de proyectos ejecutados por la Escuela de Nutrición y Dietética de la Universidad de Antioquia en la Comuna 1 Popular, se generaron contactos con líderes y lideresas que facilitaron el acercamiento al territorio, incluyendo aquellas personas que estos líderes refirieron por sus acciones importantes en el marco de las políticas de alimentación y nutrición, o por ser usuarias de los programas. También, se seleccionaron personas que implementaron programas y proyectos en esta comuna. Se realizó un muestreo teórico para la selección de los individuos y grupos, y se definió cuándo dejar de incorporar personas a partir del criterio de saturación teórica^17^. Para la recolección de información se realizaron entrevistas semiestructuradas^18^. Se construyeron guías iniciales de entrevista, que fueron ajustándose de acuerdo con la primera fase de recolección y temáticas emergentes.

Se realizaron 20 entrevistas: ocho a personas implementadoras de programas, cuatro a lideresas y ocho a personas usuarias, las cuales tuvieron una duración de 50 a 110 minutos. Para el análisis de la información, se acudió al método de la teoría fundamentada constructivista^19^ y a los tres momentos de codificación que propone el método:

Codificación abierta: se realizó un análisis línea por línea y se fueron generando categorías que permitieron ubicar los códigos en conceptualizaciones, asignando fenómenos y dimensiones debido a características que se evidenciaron como comunes, anexas o explicativas. Las primeras categorías definidas fueron: limitantes de los programas, acciones emprendidas por los participantes del estudio, fortalezas de los programas, significados de su rol, participación, desafíos y retos, reflexiones y aprendizajes. 

Codificación axial: la descripción de las subcategorías permitió empezar a definir los matices de algunos fenómenos y a identificar su importancia. 

Codificación selectiva: es un proceso que permite refinar e integrar categorías, con lo cual se logra un mayor nivel de abstracción y, por tanto, un momento en el que los hallazgos adquieren el rango de teoría^16^, lo cual derivó en cinco categorías: 1) el hambre en la Comuna 1 Popular de Medellín más allá de las cifras; 2) interacción programas-proyectos-sujetos a través mecanismos precarizantes; 3) aspectos administrativos de los programas y proyectos en el marco de un enfoque economicista; 4) acciones que simulan un ejercicio de participación; y 5) lineamientos nacionales que se perciben rígidos y descontextualizados.

Cabe señalar que el diseño del estudio tuvo en cuenta las particularidades socioculturales de las personas usuarias de los programas y de las lideresas sociales, es decir, población en situación de vulnerabilidad. La investigación consideró los principios éticos expresados en el informe Belmont de 1978, y en la Resolución 8430 de 1993 del Ministerio de Salud de Colombia, de acuerdo con la cual se clasificó el estudio como de riesgo mínimo^20^ y fue aprobada por el Comité de Ética en investigación de la Facultad de Enfermería de la Universidad de Antioquia, mediante el Acta No. 31 de 2022, previo al inicio del trabajo de campo. 

Sobre la consideración del principio de autonomía, las personas participantes firmaron un consentimiento informado en el que se detallaron aspectos como los objetivos y características del estudio, la voluntariedad para la participación, y los riesgos y beneficios asociados con esta. Se detallaban también los compromisos del equipo de investigación, incluyendo la garantía de confidencialidad y privacidad de quienes decidieron hacer parte del proceso investigativo. Para garantizar estos principios, durante el trabajo de campo se cuidó la selección de los espacios para la realización de las entrevistas y durante el estudio se tomaron medidas de seguridad frente al manejo de los datos sociodemográficos, las grabaciones de voz, las transcripciones y los consentimientos informados, que fueron resguardados con contraseñas y a los cuales solo tuvo acceso el equipo de investigación. En función del respeto por la confidencialidad, también se hizo uso de códigos para identificar a las personas participantes. Estos códigos se conformaron de la siguiente manera: la primera letra hace alusión al tipo de entrevistado (L: lideresa social, P: persona implementadora, T: persona usuaria de los programas), las dos letras siguientes corresponden a las iniciales del seudónimo con el que se identificó a cada persona, y un dígito que hizo referencia al número de la entrevista.

## RESULTADOS

### El hambre en la Comuna 1 Popular de Medellín: más allá de las cifras

Durante las conversaciones se puso de manifiesto que las dificultades para acceder a los alimentos en el territorio han estado presentes desde antes de la pandemia por covid-19 y se recrudecieron durante esta; resaltando que las dificultades no habían cesado, debido al incremento en el costo de los alimentos que, para el momento de las conversaciones con las personas participantes del estudio, se presentaban por diversas causas tanto internas como de la geopolítica mundial, como la guerra Rusia-Ucrania, el aumento del precio de los insumos agrícolas, la inflación mundial, entre otros. Así lo expresó una de las participantes del estudio:

*…la comida saludable después de pandemia. Es que eso también subió de precio y la verdad también es que el costo de la canasta familiar está jodido…* (LEM1)

Desde la visión de los implementadores, la comuna 1 Popular presenta muchas vulnerabilidades socioeconómicas. En este territorio identifican más casos de riesgo de desnutrición que en otros lugares de la ciudad donde también trabajan, y consideran que la mayoría de las familias no puede garantizar su alimentación ni en cantidad, ni en calidad. Frente a esta situación las personas implementadoras expresaron:

*De todas las sedes donde estoy, donde hay más hambre es en Santo Domingo Savio* [barrio de la Comuna 1]. *En esta comuna, las familias piden ir por la comida, si el niño no puede asistir* [a la escuela]. (PPC1)

Las descripciones expuestas evidencian la importancia que tienen los programas alimentarios y nutricionales en este territorio, y que la problemática no se resuelve con la atención específica dentro de estos, ya que el hambre traspasa las paredes del sitio de funcionamiento de los programas y es un continuo en los hogares y para todos sus miembros. 

### Interacción programas-proyectos-sujetos a través mecanismos precarizantes

Se identificó que la interacción de la política, programas o proyectos en alimentación y nutrición con los actores participantes del estudio se da a través de la precarización, lo cual se evidencia, principalmente, mediante la homogenización en la atención alimentaria y nutricional de las personas usuarias, las cuales no parecen ser la prioridad del accionar de la política. Esto se evidencia por el hecho de que los programas alimentarios y nutricionales atienden de manera diferencial a los distintos grupos poblacionales. Existe una focalización hacia las gestantes y niños y niñas menores de cinco años, mientras que para el resto de la población se observa una atención nutricional homogenizada, que entrega paquetes alimentarios o raciones de alimentos con productos ultraprocesados, sin considerar una alimentación saludable para el curso de vida. En contraste, las madres usuarias de programas o proyectos alimentarios y nutricionales para gestantes o niños y niñas menores de cinco años perciben como adecuada, variada y suficiente la alimentación que ofrecen los programas dirigidos a estos grupos poblacionales, como se evidencia en expresiones como:

[Durante la pandemia] *Les dieron muy buen paquete alimentario a los niños* […] *Era un muy buen paquete de alimentación, muy variado. Les dieron frutas, verduras, carne. Siempre es más variadito.* (LTF2)

Otro panorama parece dibujarse cuando empieza a hablarse de programas para las personas mayores de cinco años, como es el caso del Programa de Alimentación Escolar (PAE), el cual tiene cobertura a nivel nacional y cuya gestión es causa de constantes denuncias por los diversos actores. Los padres y cuidadores de niñas y niños usuarios de este programa indicaron que evidenciaban una disminución de la cantidad de cupos para la modalidad de raciones de alimentos preparados, como es el caso de los almuerzos, y que en su lugar se habían incrementado las raciones industrializadas. Desde el programa se indica que las raciones industrializadas son entregadas en instituciones que, según los lineamientos, no cuentan con las condiciones locativas que exige el programa para considerarlas como aptas para la preparación de alimentos frescos y naturales. Estas raciones industrializadas, generalmente, están constituidas por una bebida láctea procesada o ultraprocesada, una porción de fruta y una porción de harina. 

Respecto a la disminución de la oferta de preparaciones frescas y elaboradas con alimentos naturales o mínimamente procesados, se identificaron testimonios como:

*A mi hija le gustaba cuando era arroz, fríjoles, tajada, huevo, pero ahora esos pastelitos no le gustan.* (LRC3)

Vale aclarar que los alimentos naturales “son de origen vegetal o animal, inmediatamente después de la recolección, cosecha o sacrificio” y los alimentos mínimamente procesados “han sido alterados sin que se les agregue sustancias”^21^.

Diversos implementadores mencionaron el alto contenido de azúcar de ciertos productos comestibles entregados en algunos de los programas y proyectos. Los implementadores identifican en los niños y las niñas que consumen la ración industrializada, percepciones asociadas a las altas cantidades de azúcar, como lo expresa una de ellas:

*Aunque a muchos niños les gusta, otros niños se quejan mucho porque tiene mucho dulce. Ellos dicen: “es que me dan un yogurt, con un brownie, entonces es dulce con dulce”*. (PCE1)

En este mismo sentido, los adultos mayores concuerdan en describir que los paquetes alimentarios que les entregan son poco variados, no incluyen nunca alimentos frescos, pero sí productos como azúcar o chocolate con azúcar, que la mayoría de estas personas no pueden consumir por sus morbilidades preexistentes. Sin embargo, mencionan que el paquete también contiene leguminosas, aceite, café y huevos. Una de las lideresas mencionó:

*Siempre echan mucha harina, unas veces les echan arepaharina. El año pasado les estaban dando un kilo que azúcar o dos, panela… son cosas que, a la hora de comer, ni los adultos, ni los niños las pueden consumir.* (LEM1)

Algunos de los adultos mayores hicieron referencia a la entrega de bonos alimentarios hace algunos años, argumentando que los operadores que contrataban no cumplían con todas las condiciones pactadas. Evidenciaban una distinción entre la calidad de los productos que eran para la venta respecto a los productos para redimir con bonos y, además, la entrega se hacía en algunos en sitios específicos, que para muchos estaban distantes de sus lugares de residencia. 

Todos los adultos mayores usuarios indicaron que disfrutaban y aprovechaban el programa al que asistían antes de la pandemia, debido a que les suministraban alimentos preparados y así consumían una alimentación mucho más variada. Adicionalmente, podían desarrollar diversas actividades como pintura, danza, entre otros, y socializar con otras personas; sin embargo, y desconociendo las razones de esto, el programa dejó de funcionar. Al respecto uno de los usuarios indicó:

*Fue un balde de agua para todos cuando el comedor no siguió… disfrutábamos como niños, era como un fresquito*. (TMA2)

Las personas consideradas “de segunda categoría” deben competir por los recursos, ya que no están disponibles para quienes los requieren. Consecuentemente, se invierte gran cantidad de tiempo y recursos en la verificación de las condicionalidades, que le permite a la institucionalidad identificar a las personas más precarizadas como merecedoras del acceso a programas o proyectos. En esta vía, busca afinar cada vez más los mecanismos de identificación que instituye en su búsqueda de asignación de rótulos, generando diversas versiones de instrumentos como el Sistema de Identificación de Potenciales Beneficiarios de Programas Sociales (SISBEN), percibido por las personas usuarias como una clasificación que en un período las puede beneficiar, pero en otros las “perjudica” y las saca del programa al que pertenecen, por lo que representa un mecanismo de focalización que los confronta con el resto de los ciudadanos, vecinos e incluso familiares. Una de las lideresas expresó al respecto:

*…Como el SISBEN cambió, a muchas personas de la comuna les ha afectado el puntaje, porque definitivamente uno los ve que viven en un ranchito y para lo de los mercados no les da el puntaje.* (LTF2)

Este dispositivo implica, para las personas usuarias, la aceptación sin mayores reclamos. El que sean agradecidas es una expectativa de los programas que gobiernan desde la precariedad, la espera e incertidumbre constante son una combinación “perfecta” para producir personas merecedoras, quienes adicionalmente consumen los alimentos que determina el Estado, independientemente de los efectos que esto tenga para su salud, dignidad y cultura alimentaria. 

### Aspectos administrativos de los programas y proyectos en el marco de un enfoque economicista

Los aspectos administrativos de las políticas, programas y proyectos generan limitaciones en su implementación y pueden condicionar la configuración de ciertas subjetividades, principalmente en personas usuarias e implementadoras.

De manera general, las personas implementadoras expresan que los programas tienen un requerimiento excesivo de documentación, que ocupa una parte importante de su tiempo y que, dada la premura, puede diligenciarse sin necesariamente cumplir con todas las condiciones de funcionamiento. Refirieron que hay una desarticulación entre las acciones que se desarrollan en campo y lo que se registra, y que dicha información no siempre representa la realidad del programa o proyecto. El modelo de algunos programas alimentarios y nutricionales se caracteriza por una carga excesiva de diligenciamiento de formatos y documentos, que limita el contacto con las personas usuarias o estar en el territorio, como lo expresó una implementadora:

*Yo tengo que dejar tirado el trabajo de campo y el servicio se descuida muchísimo, y hay una diferencia abismal cuando yo estoy pendiente del servicio a cuando no puedo estar, porque tengo que estar metida en la oficina tratando de hacer el papeleo, porque en el papel es precioso: “ese programa es lo mejor”, porque nosotras tratamos de sacar la papelería full.* (PPC2)

En este sentido, estos aspectos administrativos, desde los relatos de los implementadores, se han tornado una barrera para la atención cercana a las personas usuarias de los programas y proyectos, así como para la prestación del servicio con la calidad que esperarían.

En este escenario, de manera general, las personas implementadoras y lideresas consideran el presupuesto como un limitante importante para la implementación de las políticas, programas y proyectos alimentarios y nutricionales, bien sea para generar una cobertura total en los casos que se requiera o para el desarrollo mínimo de acciones como adecuación, mantenimiento o generación de infraestructura, o para la dotación de los lugares o la contratación de más personal. Al respecto, una de las implementadoras expresó:

*Lo negativo de los programas es el condicionamiento a la disponibilidad de recursos por parte de la administración… Uno quisiera aumentarles cobertura, pero no hay mayor presupuesto para atención*. (PDP3)

### Acciones que simulan un ejercicio de participación

Uno de los principales efectos del entramado descrito en los apartados anteriores, se evidencia en la participación de los diferentes actores. Se trata de una participación con baja incidencia en los diferentes escenarios del diseño, implementación o seguimiento de la política, programas o proyectos. En sus relatos, las lideresas y las personas usuarias e implementadoras plantean que su participación no tiene influencia en diversos espacios. En esta línea, varias lideresas indicaron que “se aburrieron” de asistir a la Mesa de Seguridad Alimentaria de la comuna, porque no han sentido que se generen avances ni discusiones con efectos en la planeación o implementación de la política, como se evidencia a continuación:

*…yo participaba y dejé de ir porque me cansé, no quiero estar en esos espacios que son la repetición de la repetidera.* (LRC3)

Otro ejemplo de esto son los Comités de Alimentación Escolar, de los cuales consideran que no se gestionan o tramitan asuntos expuestos por las personas que habitan la comuna, ni de los niños, niñas y adolescentes que hacen parte de esta:

*…pero no, no ha pasado, por ejemplo, lo de restaurantes en la comuna, como tal en la comuna si le han hecho mucho seguimiento, pero no ha pasado nada.* (LTF2)

Para el caso específico de las lideresas, ellas no son ajenas a las dinámicas de participación en Colombia, donde el hecho de levantar la voz o liderar reflexiones y acciones, por ejemplo, en asuntos ambientales, las pueden convertir en un blanco de agresiones y, consecuentemente, hay temor a la participación reivindicativa, como lo expresaba una de ellas:

*…pero entonces es hasta un proyecto que le hace dar a uno miedo, eso de que me graben y me hagan una entrevista no lo voy a volver a hacer.* (TLA1)

Un panorama similar al descrito se presenta en las personas usuarias de programas y proyectos, especialmente quienes consideran que los procesos deben mejorar o modificarse por completo. Se configuran en personas que desconocen los procesos, en sujetos que reclaman en pocas ocasiones, que acatan las condiciones de implementación de estos programas y proyectos. Esto puede evidenciarse desde diferentes voces:

*…y viendo que es un apoyo y que uno sabe que igual eso viene de impuestos que se invierten ahí, entonces uno no le pone peros... hay que entender que la gente es agradecida y no se pone a pelear para que lo saquen*. (LEM1)

En el caso de los adultos mayores, el desconocimiento de los procesos de pertenencia al programa o proyecto es generalizado, ignoran por qué cambia la modalidad de atención o no saben si el siguiente año tendría continuidad, así como tampoco están informados acerca del inicio de operación y su duración. También refieren que no son considerados en las decisiones relacionadas con los componentes de los paquetes alimentarios. Si bien estos procesos son generadores de incertidumbre se puede notar, de manera general, una espera desde la resignación. Al respecto una de las usuarias señaló:

*…para este año no se sabe, toca esperar, porque puede que nos den o puede que cambien y les den a otros… Me tocó volverme a inscribir porque no salí favorecida, muchas no quedamos favorecidas entonces me dijeron que esperara… aunque ahora sí nos dijeron que de pronto nos llamaban para volvernos a inscribir.* (TMA2)

Por su parte, diversas personas implementadoras consideran que la comunicación y la participación en las políticas alimentarias y nutricionales en las que trabajan tienen una sola dirección, no evidencian espacios de retroalimentación directa con el programa para el mejoramiento de la implementación, resaltando que son quienes viven directamente las políticas y tienen mucho por aportar, al punto de que algunos implementadores plantearon que los espacios definidos para la participación no son reales:

*…ellos nos sientan en un auditorio a escuchar lo que ellos tienen que decir y rápido porque: “nos van a cerrar el lugar”, y esa es la excusa siempre y nosotros ya sabemos que lo utilizan como una estrategia para no escucharnos* […] *pasan las diapositivas, se demoran y se detienen en un tema y se acabó el tiempo, entonces no nos escuchan.* (PPC1)

De acuerdo con lo indicado, la comunicación no es directa con el programa, perciben artimañas y estrategias para simular la participación, acudiendo a figuras como asistencias técnicas o enlaces virtuales para dejar comentarios, las cuales no responden a las necesidades, intereses y propuestas de las personas implementadoras, ya que las sugerencias quedan en actas, pero no son respondidas ni acogidas. Uno de los implementadores lo narró de la siguiente manera:

*Nosotros con el programa como tal no tenemos comunicación directa, ellos dicen que para eso tenemos el espacio de las asistencias técnicas, pero eso es mentira… Siento que nos dan pañitos de agua tibia.* (PPC2)

Estas características coartan y coaccionan las posibilidades de participación, moldeando un tipo de *ciudadano al margen*, *receptor pasivo,* que las políticas ayudan a configurar a partir del modo de relacionamiento con la institucionalidad que las establecen, desde la verticalidad en la participación y toma de decisiones. Es difícil pensar que estos sujetos pueden concebir otras alternativas de solución a las problemáticas alimentarias y nutricionales, pues les resulta complejo considerar intervenciones que no sean de corte asistencialista cuando han sido configurados para estas. 

### Lineamientos nacionales que se perciben rígidos y descontextualizados

Las personas que implementan programas pertenecientes a políticas del orden nacional se encuentran limitadas para proponer acciones frente a asuntos como la variedad de alimentos y los ajustes de las minutas para la alimentación ya que, como se ha señalado, los lineamientos vienen determinados para todas las regiones del país, aun cuando la ciudad realiza aportes económicos adicionales para el funcionamiento del programa. Este aspecto dificulta pensar en ajustes respecto a características socioculturales diferenciales, como las de la población afrodescendiente que habita en la comuna 1 Popular. Una de las estrategias que se ha generado es que las personas que implementan el programa conforman un grupo de estudio, que incluye a técnicos de diversas áreas, quienes realizan el seguimiento a los ciclos del menú. En ese espacio retoman evaluaciones de las personas usuarias y aspectos tratados en los comités a los que asisten personas de la comunidad. No obstante, manifestaron que son pocos los ajustes que pueden realizar para dar cumplimiento a la entidad nacional que vigila la implementación. Respecto a esto, uno de los implementadores indicó:

*…pero la minuta está planteada de esa manera, pues, esos ciclos de menú están planteados así porque vienen unas directrices de la UAPA* [Unidad Administrativa Especial de Alimentación Escolar], *la Unidad de Atención de Alimentos y del MEN* [Ministerio de Educación Nacional], *y por eso no los podemos ajustar.* (PCE1)

Esta situación puede limitar la capacidad de agencia de quienes implementan los programas frente a la política, debido a que no pueden desplegar sus expectativas profesionales y las orientaciones de su formación a favor de las personas usuarias de los programas, disminuyendo sus posibilidades de acción. Este recorrido permite identificar los sentimientos de frustración profesional en las personas que implementan los programas, a partir de su deseo de desarrollar su trabajo de la mejor manera posible y en beneficio de la población; sin embargo, se encuentran limitadas en su accionar por las condiciones técnicas del orden nacional a las que deben alinearse.

## DISCUSIÓN

Asistimos a un orden global cuyas transformaciones afectan casi cualquier aspecto de la vida de los sujetos, quienes, independientemente del lugar en el que se encuentren, sienten sus efectos^10^. En una sociedad, con crecientes preocupaciones por la seguridad, emerge la paradoja de las profundas inseguridades vitales que deben ser asumidas por los sujetos. Se trata de un aspecto que se constituye en parte importante de la realidad actual y, siguiendo a Castel, configura, en buena medida, la experiencia social. En este marco, las subjetividades precarizadas se forman a partir de la incertidumbre. El principio general civilizatorio en la sociedad moderna no es el progreso social, sino la incertidumbre, “hacer de la inseguridad el horizonte insuperable de la condición del hombre moderno”^4^. Se trata de subjetividades con una marcada tendencia neoliberal, contexto en el cual la precarización se ha convertido en un instrumento de gobierno, al servicio de la regulación y el control social. Es así como Lorey establece que la precarización es una “técnica de manejo mínimo del umbral de la vulnerabilidad social que es apenas tolerable”^22^, que produce inseguridad en las personas, al estar expuestas constantemente a la contingencia en todas las esferas de la vida (laboral, económica, social). Además, existe una pretensión de normalización de estas circunstancias. 

Consecuentemente, es relevante reconocer que la vida de las personas es valorada en función de su instrumentalización para intereses económicos, para lo cual el poder hegemónico toma una posición de uso y dominio de las vidas^23^. Se produce un entramado que funciona de manera invisible y va naturalizando las ideas dominantes, intentando alcanzar dependencias simbólicas, lo cual se considera una novedad en el neoliberalismo, dada su capacidad de producir subjetividades desde un paradigma empresarial, individualista, competitivo y ahora precarizante, a un nivel en el que se considera que “el botín de guerra actual del capitalismo es la subjetividad”^23^. 

La producción de subjetividades por las lógicas del poder se presenta a través de diferentes figuras: la vida como una empresa, el empresarismo de sí, la promoción excesiva de la figura del emprendedor, el relacionamiento de la vida bajo las formas de la mercancía/mercado, la competencia constante a través de intereses individuales y la precarización^23^^,^^24^^,^^25^. Se acude a mecanismos que promueven diversos tipos de subjetividades, en cuanto el neoliberalismo “necesita producir un hombre nuevo, no reclamado por ninguna causa o legado simbólico y precario, líquido, fluido y volátil como la propia mercancía”^23^. Siguiendo a Astete y Vaccari^25^, y en correspondencia con los hallazgos de este estudio, uno de los tipos de subjetividades producidas, son las “subjetividades agradecidas”, aquellas donde “lo que se recibe no se aprecia como derecho sino como acto de gracia”^25^. 

El carácter relacional e institucional de la vida humana implica la configuración no solo del sujeto y de sus diferentes momentos interactivos, sino también de los espacios sociales en los que esas relaciones se producen. Los diferentes espacios de una sociedad concreta están estrechamente relacionados entre sí y tienen implicaciones subjetivas^26^; aquí, los escenarios de las políticas alimentarias y nutricionales se incluyen como espacio de formación de subjetividades. 

Dentro de los procesos de precarización, la angustia está presente; se produce en un ámbito en el que las personas deben sentir que todo está puesto en duda, en el que nadie sabe cuánto va a durar, por ejemplo, en un trabajo^23^ y, en este caso, por cuánto tiempo se es merecedor de pertenecer a un programa alimentario y nutricional o cuánto tiempo durará un proyecto en el territorio. Es llamativo que, a pesar de contar con gran cantidad de personal dedicado a las focalizaciones y a afinar sus instrumentos, una frase de Jorge Alemán puede describir la angustia de los adultos mayores participantes de este estudio al no tener información y, mucho menos, ser consultados acerca de la implementación de los programas alimentarios y nutricionales en su territorio: la angustia “siempre se da desde un lugar de desamparo […] hay una soledad radical, aunque sean muchos los que intervengan”^23^. El individuo debe hacer frente, solitariamente, a los riesgos vitales; en consecuencia, es a este a quien le corresponde, sí puede, su propio aseguramiento^4^.

Así, en esta arena de juego, la individualidad es un factor de importancia central. Las políticas, programas y proyectos alimentarios y nutricionales se tornan instrumentos de gobierno, esto es, pueden conducir conductas como forma de administrar los cuerpos, y es más fácil actuar sobre el comportamiento de las personas desde su individualidad^22^. Esto allana también el camino para la competencia que deben realizar las personas pertenecientes a los grupos menos valorados de la sociedad, con el objetivo de acceder a uno de los cupos de estos programas alimentarios y nutricionales, demostrando mayor necesidad que el resto de las personas que habitan su barrio, comuna o ciudad. Una lucha individual por el merecimiento. Una disputa por el cupo. Esto refuerza la competencia, ideal del relacionamiento neoliberal, desarrollando procesos de participación funcionales al programa, que no trabajan en torno a causas más estructurales de las problemáticas sociales^8^. Aguirre^27^ señala que resulta difícil pensar en la participación de los actores involucrados cuando un programa ya está diseñado, ya tiene un presupuesto sin posibilidad de ajuste o movilidad interna, las actividades y funciones ya están claramente definidas y la única opción es aceptar o rechazar hacer parte de ello. 

En esta lógica neoliberal, los individuos tienen que competir y se deben mostrar con una mejor dotación de capital humano, lo cual deriva en que, dentro de la sociedad, exista un grupo de personas con una mejor atención, con acciones preferenciales que reflejan más recursos y mejor servicio para la población susceptible de formar capital humano como un factor de producción en una economía de mercado, dando todas las garantías al capital. Así, se producen subjetividades en cuanto cada individuo “tiene que construir su propia historia e interpretar su propio pasado […] no debe preocuparse por los logros de los demás, ni los fines colectivos de la sociedad”, el sujeto está concentrado buscando su reducido bienestar, que dificulta encontrar sentido en la solidaridad^8^, rompiendo la identidad social como producto de la “memoria colectiva”^26^.

No obstante, lo que nos muestra el caso de estudio es que no necesariamente el objeto de la competencia que promueve el neoliberalismo está asociado a ser el más fuerte en una lógica de mercado, sino también a ser quien cuente con las condiciones para merecer la recepción de un servicio. Lo central aquí, consecuentemente, no es el objeto de la competencia, sino la competencia en sí misma, porque desdibuja posibilidades de vivir solidariamente y encumbra el ideal del individualismo que es funcional a esta racionalidad. 

Por otro lado, es reconocido que las políticas públicas pueden configurarse en formas de biopoder, biologizando procesos culturales y sociales. En el caso de las políticas públicas alimentarias y nutricionales ese proceso biologizante se relaciona con su basamento, principalmente, en conocimientos científico-nutricionales procedentes del saber biomédico, el cual requiere definir un ser humano “normal”, usado como parámetro para comparar al resto de las personas^28^, modelando subjetividades y generando marcos de acción sobre aquellos que no cumplen con un peso y talla ideal. 

En la colonización de las subjetividades por parte del biopoder, el estándar de la normalidad y la alimentación juegan un papel muy útil a las características que debe tener una población apta para el mercado; se biologizan procesos culturales, con una institucionalidad que ha definido *qué deben* comer las personas. Este patrón de normalidad termina por asignar responsabilidades individuales sobre el estado nutricional, reduciendo todo lo que allí confluye a malas decisiones de los sujetos. Para Pemjeam^28^ se trata de un dispositivo erigido como un mecanismo disciplinario, el cual hace su aporte a “la resignación de los sujetos a lo existente” o a lo que él llama una “movilidad replegada”. 

Ahora bien, el sujeto moderno debió formarse urbano y con un cuerpo productivo, que favoreciera la búsqueda del “desarrollo”, evidenciando que “la relación entre cuerpo y sujeto está atravesada por el poder; así, las formas de este intentan cuerpos vinculados a los ‘tipos’ de sujetos que se privilegia en una sociedad”^29^. Esta situación lleva, sin duda, a preguntarse por posturas que parecieran antagónicas al hablar de individualización y homogenización, pero no son asuntos opuestos, debido a que como lo expresa Foucault:

No se toma al individuo en el nivel del detalle sino, al contrario, mediante mecanismos globales, de tal manera que se obtengan estados globales de equilibrio y regularidad; en síntesis, de tomar en cuenta la vida, los procesos biológicos del hombre/especie y asegurar en ellos no una disciplina, sino una regularización.^5^

Es claro que, ante la creciente producción, distribución y publicidad de productos alimentarios ultraprocesados, el papel de las personas que implementan programas para orientar y acompañar recomendaciones de alimentación es vital, pero no puede desconocerse que este tipo de orientaciones han sido instrumentalizadas, tomadas como estrategia de conducción de los sujetos^5^. 

Es clave señalar que las políticas públicas y sus instrumentos tienen un “referencial” de acuerdo con sus interpretaciones de los problemas públicos y las formas de tramitarlos. En lo que respecta a las políticas públicas alimentarias y nutricionales, el referencial de alimentos por el mercado se basa en que la oferta debe establecerse a escala mundial, aprovechando las ventajas comparativas de cada país^30^, lo cual ha facilitado el monopolio de las grandes industrias en el suministro global de alimentos^31^. 

## CONCLUSIONES

De acuerdo con los hallazgos de este estudio, aunque hay diversos significados y formas de posicionarse frente a la política, programas y proyectos en alimentación y nutrición, el principal fenómeno emergido de los datos es que las políticas públicas alimentarias y nutricionales configuran subjetividades precarizadas a través de mecanismos como la homogenización en la atención alimentaria y nutricional de los ciudadanos. Asimismo, los aspectos administrativos de los programas y proyectos limitan el desarrollo de acciones acordes con las necesidades de las personas usuarias y las intencionalidades de quienes implementan los programas, y generan una participación con baja incidencia política. Sin embargo, algunos actores, principalmente las lideresas sociales, promueven acciones para “paliar” el hambre y fomentan el acceso a la información por parte de las personas usuarias y el desarrollo de acciones que movilicen ajustes en el funcionamiento de las políticas y programas.

Se evidenció de manera más generalizada que las acciones desarrolladas en el marco de las políticas públicas alimentarias y nutricionales limitan y coartan las posibilidades de acción, buscando una identificación con las estrategias de mitigación, merecimiento y espera sin reclamos. Adicionalmente, la visión y alienación de sujetos individualizados restringen las posibilidades de acción colectiva en un panorama en el que las políticas públicas pueden operar como dispositivos que conciben a los sujetos como receptores pasivos, que a menudo son desconocidos en el proceso del ciclo de vida de las políticas. Así, estas interacciones entre los sujetos y las políticas públicas pueden configurar un sujeto receptor, que puede empezar a determinarse a través de mecanismos regularizadores que buscan de él un comportamiento específico para hacerse merecedor, que le permita ser objeto de las intervenciones estatales; sin embargo y a pesar de esta intencionalidad, también surgen interpelaciones y acciones que buscan controvertir el papel asignado. 

Cabe señalar que usualmente se ha privilegiado una mirada biomédica de la nutrición, de lo cual se deriva su carácter vertical, que no responde a los contextos ni prácticas de las comunidades. Este trabajo insta a la inclusión de la dimensión de las subjetividades, a ubicar a los sujetos en el centro de las políticas, quienes viven y construyen alrededor de lo alimentario y nutricional, y cuya participación real contribuiría a un diseño, implementación y evaluación de políticas más pertinentes y situadas.

Actualmente, las políticas públicas alimentarias y nutricionales priorizan a la primera infancia y el resto de la población es homogenizada en su atención nutricional, generando diferenciaciones en la garantía del derecho humano a la alimentación y favoreciendo el discurso de la formación de capital humano; adicionalmente, las acciones de mitigación sobre el hambre y las problemáticas alimentarias responden a las lógicas de compensación y mitigación que sustentan los sistemas de protección social desde inicios de la década de 1990. A partir de allí, las múltiples comprensiones sobre la alimentación se reflejan en las tensiones y disensiones entre los actores que participan en la construcción de las políticas alimentarias y nutricionales. 

Es difícil pensar que los sujetos pueden concebir otras alternativas de solución a las problemáticas alimentarias y nutricionales, pues les resulta complejo considerar intervenciones que no sean de corte asistencialista cuando han sido subjetivados para estas. Adicionalmente, estas situaciones pueden limitar la capacidad de agencia de quienes implementan los programas frente a la política, debido a que no pueden desplegar sus expectativas profesionales y las orientaciones de su formación a favor de las personas usuarias de los programas, disminuyendo sus posibilidades de acción.

En suma, la implementación de políticas alimentarias y nutricionales se convierte en un contexto de relación entre el Estado y la sociedad, del que deriva una convergencia de estrategias asistencialistas tendientes a instrumentalizar, de manera particular, la potencialidad de la participación social, inmersa en escenarios de traspaso de funciones estatales al sector privado y en las que las comunidades tienden a ser “subsumidas” en las responsabilidades que se les asignan desde las políticas públicas^32^.

De las conclusiones de este estudio pueden derivarse algunas recomendaciones: las políticas, programas y proyectos alimentarios y nutricionales deben ubicar al sujeto en el centro de la formulación, implementación y evaluación de estos instrumentos, lo cual implica enfocar los procesos en construcciones que tengan en cuenta las necesidades y las capacidades de las personas involucradas. Si bien los indicadores antropométricos han sido y serán incluidos por su importancia como información de referencia, deben considerarse las subjetividades alrededor de las prácticas alimentarias, que están cargadas de expectativas, acciones y, en muchos casos, tradiciones. 

La justicia alimentaria puede configurarse en una forma de ubicar al sujeto en el centro de la política (usuarios de programas y proyectos, implementadores y productores de alimentos), en cuanto implica más que una mera acción distributiva, y requiere, además, el reconocimiento de la voz política y la representación de todas las personas en la toma de decisiones y las políticas públicas sobre producción, consumo y distribución de alimentos, así como las conexiones normativas entre las prácticas relacionadas con los alimentos y la autodeterminación colectiva^33^.

Por otro lado, es necesario priorizar la inversión de recursos en infraestructura que permita la preparación de alimentos frescos y saludables, por encima de la adición de micronutrientes a algunos alimentos y limitando el tiempo de implementación de raciones industrializadas a causa de la falta de infraestructura. Las políticas y programas del orden nacional deben implementar modificaciones que permitan los ajustes necesarios en las regiones del país, considerando la cultura alimentaria como eje fundamental de una alimentación variada, saludable y sustentable y priorizando la disminución de productos ultraprocesados de minutas y paquetes alimentarios.

Finalmente, cabe mencionar que la principal limitación de este estudio se centra en la imposibilidad de haber realizado observación participante en la implementación de los programas o proyectos analizados.
